# 2’-Fluoro-2’-deoxycytidine inhibits murine norovirus replication and synergizes MPA, ribavirin and T705

**DOI:** 10.1007/s00705-020-04759-4

**Published:** 2020-08-08

**Authors:** Peifa Yu, Yining Wang, Yunlong Li, Yang Li, Zhijiang Miao, Maikel P. Peppelenbosch, Qiuwei Pan

**Affiliations:** Department of Gastroenterology and Hepatology, Erasmus MC-University Medical Center, Room Na-1005, ‘s-Gravendijkwal 230, 3015 CE Rotterdam, The Netherlands

## Abstract

Noroviruses are the main causative agents of acute viral gastroenteritis worldwide. However, no vaccine or specific antiviral treatment is available, imposing a heavy global health burden. The nucleoside analogue 2’-fluoro-2’-deoxycytidine (2’-FdC) has been reported to have broad antiviral activity. Here, we report that 2’-FdC significantly inhibits murine norovirus replication in macrophages. This effect was partially reversed by exogenous supplementation of cytidine triphosphate. The combination of 2’-FdC with mycophenolic acid, ribavirin or favipiravir (T705) exerts synergistic antiviral effects. These results indicate that 2’-FdC is a potential candidate for antiviral drug development against norovirus infection.

**Electronic supplementary material:**

The online version of this article (10.1007/s00705-020-04759-4) contains supplementary material, which is available to authorized users.

## Introduction

Human norovirus (HuNV) is a non-enveloped, positive single-stranded RNA virus [1]. Recently, noroviruses have been classified into at least 10 genogroups (GI-GX) on the basis of the amino acid sequence diversity of the viral VP1 protein [2]. Viruses in the GI, GII, GIV, GVIII and GIX genogroups can infect humans and are a major cause of acute epidemic viral gastroenteritis worldwide [2]. It is estimated that noroviruses are responsible for 699 million gastroenteritis cases per year [3] and 200,000 deaths in children under 5 years of age in developing countries [4]. Although norovirus gastroenteritis is usually self-limiting, it has been recognized as an emerging burden in immunocompromised populations, particularly transplant recipients [5, 6]. However, research into HuNV infection has been hampered by the lack of availability of robust experimental models sustaining viral infection. Murine norovirus (MNV), which is capable of replicating in both cell culture and small-animal models, shares similar traits with HuNV in structural and genetic features and has thus been widely used as a surrogate model [7, 8]. To date, no vaccine or specific antiviral treatment is available, and clinical management is restricted to supportive care and oral rehydration. Thus, the development of specific antiviral drugs for norovirus infection is urgently needed.

Potential inhibitors of noroviruses have been identified, and some of these have demonstrated efficacy in experimental models. Ribavirin has been extensively studied and exhibits broad antiviral activity against multiple viruses, including hepatitis C virus (HCV) [9], hemorrhagic fever virus [10], hepatitis E virus [11, 12], and norovirus [13]. In a clinical study, ribavirin treatment resulted in complete viral clearance in a subset of norovirus-infected patients, but treatment failure occurred in two cases [14]. We demonstrated previously that mycophenolic acid (MPA), a potent inhibitor of IMP dehydrogenase (IMPDH), can inhibit norovirus replication in cell culture [15]. Favipiravir, also known as T-705, has been approved for the treatment of influenza in Japan and has been repositioned to treat patients with Ebola virus infection [16, 17]. It has been shown to be effective against noroviruses, but the treatment can induce mutagenesis in mice and in patients, challenging the application of favipiravir for treating chronic norovirus infection [18].

Recently, 2’-fluoro-2’-deoxycytidine (2’-FdC), also known as 2’-deoxy-2’-fluorocytidine, has been reported to exert broad antiviral activity against HCV, Lassa virus, Crimean-Congo hemorrhagic fever virus, and bunyaviruses [19–22]. Given the success of 2’-FdC against the abovementioned viruses, we aimed to investigate the potential antiviral activity of this compound against MNV replication.

## Materials and methods

### Reagents

2’-Fluoro-2’-deoxycytidine was purchased from Biosynth Carbosynth and dissolved in dimethyl sulfoxide (DMSO, Sigma, Zwijndrecht, The Netherlands). MPA (Sigma), ribavirin (Bio-Connect BV), T705 (BioVision), cytidine triphosphate (CTP; Sigma), guanosine triphosphate (GTP; Sigma), human IFN-α (Thermo Scientific, The Netherlands) and JAK inhibitor 1 (Santa Cruz Biotechnology, USA) were used. A rabbit polyclonal antiserum against MNV NS1/2 [23] was kindly provided by Prof. Vernon K. Ward (School of Biomedical Sciences, University of Otago, New Zealand). β-actin antibody (#sc-47778) was purchased from Santa Cruz Biotechnology. IRDye® 800CW-conjugated goat anti-rabbit and goat anti-mouse IgGs (Li-Cor Bioscience, Lincoln, USA) were used as secondary antibodies, as appropriate.

### Cells and viruses

RAW264.7 and J774A.1 were cultured in Dulbecco’s modified Eagle’s medium (DMEM; Lonza Verviers, Belgium) supplemented with 10% (vol/vol) heat-inactivated fetal calf serum (FCS; Hyclone, Logan, UT, USA) and 100 μg of streptomycin, and 100 IU of penicillin per mL. The murine norovirus strain MNV-1 (MNV-1.CW1), the acutely cleared strain MNV^CW3^, and the persistent strain MNV^CR6^ were produced by consecutively inoculating the virus (kindly provided by Prof. Herbert Virgin, Department of Pathology and Immunology, Washington University School of Medicine) onto RAW264.7 cells [24]. Human Huh7 hepatocellular carcinoma cells harboring a genotype 1 HuNV replicon (HG23) were kindly provided by Dr. Kyeong-Ok Chang (Kansas State University) [25]. A neomycin resistance gene was engineered into ORF2, conferring HG23 resistance to neomycin. Gentamicin (G418; Gibco) was added to HG23 culture medium at 0.5 mg/mL for selection before experimentation.

### Tcid_50_

MNV was quantified using a 50% tissue culture infectious dose (TCID_50_) assay. Briefly, tenfold dilutions of MNV were inoculated onto RAW264.7 cells grown in a 96-well tissue culture plate at 1,000 cells/well. The plate was incubated at 37°C for another 5 days, and each well was examined under a light microscope for a cytopathic effect (CPE). The TCID_50_ was calculated by using the Reed-Muench method.

### Antiviral assay

The antiviral assay was initiated by inoculating RAW264.7 or J774A.1 cells with MNV at a multiplicity of infection (MOI) of 1. After 1 h of infection, cells were washed twice with phosphate-buffered saline (PBS) to remove free virus particles and then treated with the indicated compounds. For combination assays, RAW264.7 cells were infected with the virus for 1 h, and the medium was replaced with medium containing 2’-FdC, MPA, ribavirin, or T705, alone or in combination, at the indicated concentrations. After 20 h of treatment, total RNA, protein and the supernatant samples were collected and further analyzed by qRT-PCR, western blot and TCID_50_ assay, respectively.

### qRT-PCR

Total RNA was isolated using a Macherey NucleoSpin RNA II Kit (Bioke, Leiden, The Netherlands) and quantified using a NanoDrop ND-1000 spectrophotometer (Wilmington, DE, USA). cDNA was synthesized from 500 ng of RNA using a cDNA synthesis kit (TaKaRa Bio, Inc., Shiga, Japan). The cDNA of all target genes was quantified by SYBR-Green-based (Applied Biosystems) real-time PCR on a StepOnePlusTM System (Thermo Fisher Scientific LifeSciences) according to the manufacturer’s instructions. Human glyceraldehyde-3-phosphate dehydrogenase (GAPDH) and murine GAPDH genes were used as reference genes to normalize gene expression. The relative expression of the target gene was calculated as 2-ΔΔCT, where ΔΔCT = ΔCT_sample_ - ΔCT_control_ (ΔCT = CT_[target gene]_ - CT_[GAPDH]_). All primer sequences are listed in Supplementary Table 1.

### Western blot

Cultured cells were lysed in Laemmli sample buffer containing 0.1 M DTT, heated for 5 min at 95 °C, and loaded onto a 10% sodium dodecyl sulfate polyacrylamide gel electrophoresis (SDS-PAGE) gel. After electrophoresis, the proteins were electrophoretically transferred to a polyvinylidene difluoride (PVDF) membrane (pore size, 0.45 μM; Invitrogen) for 2 h with an electric current of 250 mA. Subsequently, the membrane was blocked with a mixture of 2.5 mL of blocking buffer (Odyssey) and 2.5 mL of PBS containing 0.05% Tween 20 for 1 h, followed by overnight incubation with primary antibodies (1:1000) at 4 °C. The membrane was washed three times and then incubated with IRDye-conjugated secondary antibody (1:5000) for 1 h. After washing three times, protein bands were detected using an Odyssey 3.0 Infrared Imaging System (Li-Cor Biosciences).

### Confocal fluorescence microscopy

RAW264.7 or J774A.1 cells infected with MNV-1 at an MOI of 1 for 1 h, and the culture medium was replaced by medium containing different concentrations of 2’-FdC in an 8-well chamber (cat. no. 80826; ibidi GmbH) for 20 h. The cells were fixed with 4% paraformaldehyde in PBS, permeablized with 0.2% Triton X-100, blocked with 5% skim milk for 1 h, reacted with rabbit polyclonal antiserum against MNV NS1/2, and stained with 4’,6-diamidino-2-phenylindole (DAPI). Secondary antibody anti-rabbit IgG (H+L), F(ab’)2 fragment (Alexa Fluor® 488 conjugate) was used. Imaging was performed on a Leica SP5 confocal microscopy using a 63x oil objective.

### IC_50_ and CC_50_ calculation

The 50% inhibitory concentration (IC_50_) value and 50% cytotoxic concentration (CC_50_) were calculated using the formula Y¼Bottom þ (Top-Bottom)/ (1 þ 10^((LogIC50-X)*HillSlope)) using GraphPad Prism 5 software (GraphPad Prism 5; GraphPad Software Inc., La Jolla, CA, USA).

### MTT assay

Cells were seeded into 96-well tissue culture plates, and cell viability was assessed by adding 10 mM 3-(4,5-dimethyl-2-thiazolyl)-2,5-diphenyl-2H-tetrazolium bromide (MTT) (Sigma, Zwijndrecht, The Netherlands). After 3 h, the medium was replaced with 100 μL of DMSO and was incubated at 37°C for 50 min. The absorbance at 490 nm was recorded using a microplate absorbance reader (Bio-Rad, CA, USA).

### Statistical analysis

Data are presented as the mean ± SEM. Comparisons between groups were performed using the Mann-Whitney test in GraphPad Prism 5.0 (GraphPad Software Inc., La Jolla, CA, USA). Differences were considered significant at a *P*-value less than 0.05.

## Results and discussion

**Fig. 1:**
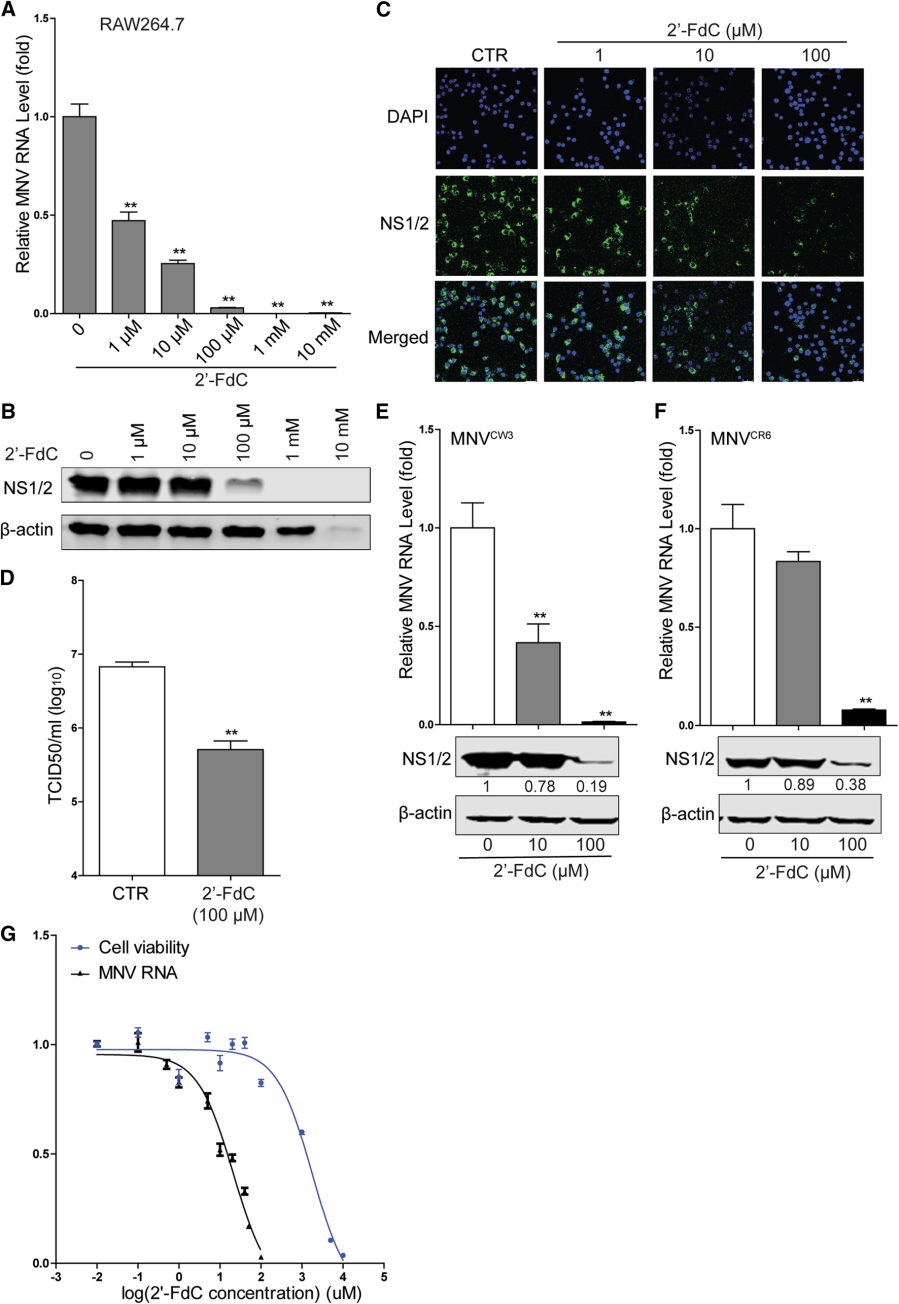
2’-FdC exerts anti-MNV activity in RAW264.7 cells. RAW264.7 cells were infected with MNV-1 at an MOI of 1 for 1 h, and the culture medium was replaced by medium containing different concentrations of 2’-FdC for 20 h. (A) The viral RNA level and (B) NS1/2 protein expression were analyzed by qRT-PCR (n = 6) and western blotting, respectively. (C) RAW264.7 cells were infected with MNV-1 at an MOI of 1 for 1 h, and the culture medium was replaced by medium containing different concentrations of 2’-FdC for 20 h. Viral NS1/2 protein expression was analyzed by confocal assay. (D) RAW264.7 cells were infected with MNV-1 at an MOI of 1 for 1 h and then left untreated or treated with 100 µM 2’-FdC for 20 h. The viral titer was determined by TCID_50_ assay (n = 6). RAW264.7 cells were infected with (E) MNV^CW3^ or (F) MNV^CR6^ at a MOI of 1 for 1 h and then left untreated or treated with 2’-FdC (10 µM and 100 µM, respectively) for 20 h. The viral RNA level and NS1/2 protein expression were analyzed by qRT-PCR (n = 6) and western blotting, respectively. (G) RAW264.7 cells were left uninfected or infected with MNV-1 at an MOI of 1 for 1 h and then left untreated or treated with different concentrations of 2’-FdC for 20 h. The 50% cytotoxic concentration (CC_50_) (n = 16) and 50% inhibitory concentration (IC_50_) (n = 4-6) against viral replication were calculated using GraphPad Prism 5 software. Data were normalized to the untreated control (set as 1). **, *P* < 0.01. β-actin was used as a loading control. For immunoblot results (E and F), the band intensity of the NS1/2 protein in each lane was quantified using Odyssey software, and the quantification results were normalized to β-actin expression (control, set as 1)

To test the potential anti-norovirus activity of 2’-FdC, we used the murine norovirus as a surrogate model. We found that 2’-FdC significantly decreased the viral RNA and NS1/2 protein expression of MNV-1 in RAW264.7 cells, a murine macrophage cell line that is susceptible to MNV propagation (Fig. 1A and B). The inhibitory effect of this compound was confirmed, with decreased viral NS1/2 expression observable by confocal fluorescence microscopy (Fig. 1C). Moreover, the viral titer was found to decrease after treatment with 100 µM 2’-FdC (Fig. 1D). To further examine the antiviral effects, another murine macrophage cell line, J774A.1, was used, and a similar inhibitory effect of 2’-FdC on MNV-1 replication was observed, with decreased viral RNA and NS1/2 protein expression (Supplementary Fig. 1).

With 2’-FdC emerging as a potential anti-MNV candidate, we further evaluated its antiviral effect on two other MNV strains with distinct biological characteristics, the acutely cleared strain MNV^CW3^ and the persistent strain MNV^CR6^. Notably, 2’-FdC inhibited viral RNA replication and protein expression of both viral strains (Fig. 1E and F). In addition, the IC_50_ value of 2’-FdC against MNV-1 replication in RAW264.7 cells was 20.92 µM (Fig. 1G), and the CC_50_ of 2’-FdC in RAW264.7 cells was 1.768 mM (Fig. 1G). Moreover, we tested the antiviral effect of 2’-FdC on HuNV by using HG23 cells harboring an HuNV replicon and found moderate inhibition of viral replication (Supplementary Fig. 2).

**Fig. 2:**
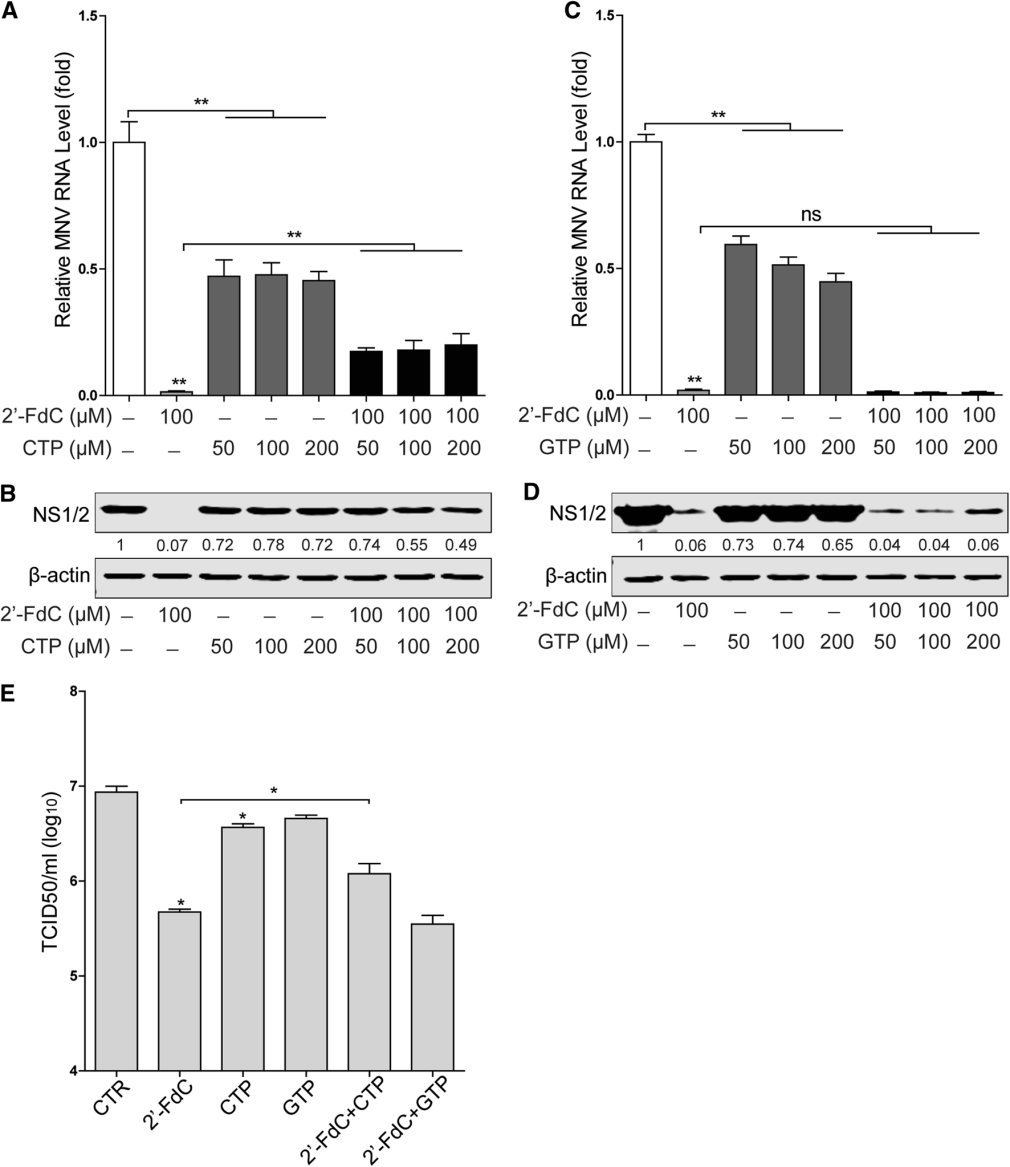
CTP, but not GTP, reverses 2’-FdC-mediated inhibition of MNV replication. RAW264.7 cells were infected with MNV-1 at an MOI of 1 for 1 h and then either left untreated or treated with 2’-FdC, CTP or combinations thereof at the indicated concentrations for 20 h. (A) Viral RNA and (B) NS1/2 protein expression were analyzed by qRT-PCR (n = 6) and western blotting, respectively. RAW264.7 cells were infected with MNV-1 at an MOI of 1 for 1 h and then left untreated or treated with 2’-FdC, GTP or combinations thereof at the indicated concentrations for 20 h. (C) Viral RNA and (D) NS1/2 protein expression were analyzed by qRT-PCR (n = 6) and western blotting, respectively. (E) RAW264.7 cells were infected with MNV-1 at an MOI of 1 for 1 h and then left untreated or treated with 2’-FdC (100 µM), CTP (100 µM), GTP (100 µM) or combinations thereof for 20 h. The viral titers were determined by TCID_50_ assay (n = 4). Data were normalized to the untreated control (set as 1). *, *P* < 0.05; **, *P* < 0.01; ns, not significant. β-actin was used as a loading control. For immunoblot results (B and D), the band intensity of the NS1/2 protein in each lane was quantified using Odyssey software, and the quantification results were normalized to β-actin expression (control, set as 1)

Nucleoside analogues have been reported to induce an antiviral interferon response [13, 26]. Interferon-stimulated genes (ISGs) are considered the ultimate effectors against viral infection, but we found that 2’-FdC treatment did not significantly increase ISG expression (Supplementary Fig. 3A), and inhibition of viral RNA production by 2’-FdC was not affected by treatment with a JAK inhibitor (Supplementary Fig. 3B), suggesting that the antiviral effect of 2’-FdC does not require ISG induction. Theoretically, nucleoside analogues exert potential antiviral activity because they bind to the viral RNA polymerase active site to impede viral replication. Since 2’-FdC is an analogue of cytidine and fluorine is isosteric with a hydroxyl group, chemical conversion of 2’-FdC to the corresponding 2’-FdC-triphosphate (FdCTP) results in a compound with antiviral activity against HCV, possibly targeting the viral NS5B enzyme [20]. Thus, we performed a competition assay by using CTP and GTP, which showed that CTP partially reversed the inhibitory effects of 2’-FdC on MNV replication, as reflected in viral RNA and protein levels (Fig. 2A and B) as well as the viral titers (Fig. 2D). In contrast, no significant effect of GTP on 2’-FdC-mediated inhibition of viral replication was observed (Fig. 2C and D). Interestingly, we found that both CTP and GTP decreased the viral RNA level and NS1/2 protein expression (Fig. 2A-D). It has been shown that MNV infection can induce viperin transcription in RAW264.7 cells [27], and viperin can convert CTP into 3’-deoxy-3’,4’-didehydro-CTP (ddhCTP), which acts as a chain terminator of RNA-dependent RNA-polymerases and inhibits replication of Zika virus [28]. Moreover, exogenous CTP/GTP might complete with the endogenous CTP/GTP for MNV replication [29]. These results suggest a potential mechanism of action of 2’-FdC against MNV, and it needs to be investigated whether 2’-FdC exerts anti-MNV activity by targeting the viral replicase.

**Fig. 3:**
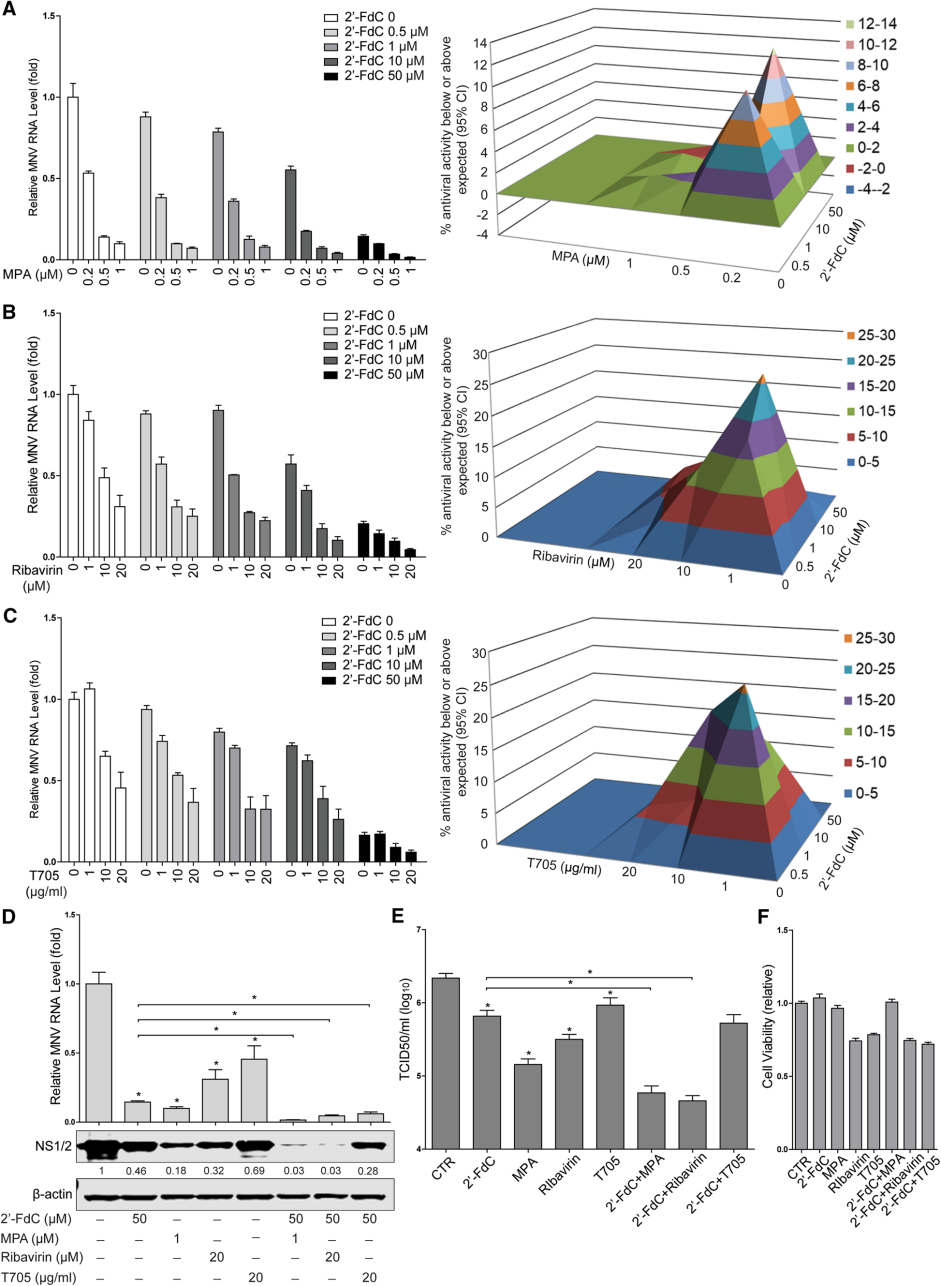
Synergistic anti-MNV effects of 2’-FdC with MPA, ribavirin or T-705. (A) RAW264.7 cells were infected with MNV-1 at an MOI of 1 for 1 h and then left untreated or treated with 2’-FdC and MPA at the indicated concentrations for 20 h, alone or in combination. The combined effect of 2’-FdC and MPA on viral replication was analyzed by using qRT-PCR assay (n = 4) and mathematical modeling using MacSynergy. (B) RAW264.7 cells were infected with MNV-1 at an MOI of 1 for 1 h and then left untreated or treated with 2’-FdC and ribavirin with the indicated concentrations for 20 h, alone or in combination. The combined effect of 2’-FdC and ribavirin on viral replication was analyzed using a qRT-PCR assay (n = 2-4) and mathematical modeling using MacSynergy. (C) RAW264.7 cells were infected with MNV-1 at an MOI of 1 for 1 h and then left untreated or treated at the 2’-FdC and T705 with indicated concentrations for 20 h, alone or in combination. The combined effect of 2’-FdC and T705 on viral replication was analyzed using a qRT-PCR assay (n = 4) and mathematical modeling using MacSynergy. The three-dimensional surface plot represents the differences (within 95% confidence interval) between actual experimental effects and theoretical additive effects of the combination at various concentrations of the two compounds. RAW264.7 cells were infected with MNV-1 at an MOI of 1 for 1 h and then left untreated or treated with 2’-FdC and MPA, ribavirin, or T705 at the indicated concentrations for 20 h, alone or in combination. (D) The viral RNA level and NS1/2 protein expression, and (E) viral titers were analyzed by qRT-PCR (n = 4; data were derived from A, B and C), western blotting, and TCID_50_ (n = 4) assays, respectively. (F) RAW264.7 cells were left untreated or treated with 2’-FdC (50 µM), MPA (1 µM), ribavirin (20 µM), T705 (20 µg/ml) or combinations thereof for 20 h. Cytotoxicity was determined by MTT assay (n = 16). *, *P* < 0.05. β-actin was used as a loading control. For immunoblot results (D), the band intensity of the NS1/2 protein in each lane was quantified using Odyssey software, and the quantification results were normalized to β-actin expression (control, set as 1)

Since MPA, ribavirin, and T705 have been reported to have anti-norovirus activity, a combined treatment using 2’-FdC together with these compounds might be envisaged. To achieve better antiviral efficacy, we evaluated the combined antiviral effects of 2’-FdC with MPA by mathematical modeling using MacSynergy [30]. Surprisingly, the results showed a moderate synergistic antiviral effect (36.57 µM^2^%), which is greater than either 2’-FdC or MPA alone (Fig. 3A). Similar synergistic antiviral effects were observed when combining 2’-FdC with ribavirin (99.18 µM^2^%) or T705 (112.47 µM^2^%) (Fig. 3B and C). To confirm the predicted synergistic antiviral effects, we measured viral protein expression and viral titers by using high concentrations of the antivirals without major cytotoxicity (Fig. 3F). As shown in Fig. 3D and E, the viral NS1/2 protein expression and viral titers were further decreased when the antivirals were used in combination, supporting the synergistic antiviral effects of 2’-FdC with MPA, ribavirin, or T705 against MNV replication.

Despite their wide clinical application, the potential side effects or unintended off-target effects of nucleoside analogues should be considered. Induction of mutagenesis by T705 treatment in patients has raised questions for treating chronic norovirus infections [18]. Previous studies have reported that 2’-FdC exhibits delayed toxicity after prolonged exposure, and no adverse clinical effects were observed in rats and woodchucks after 90 days of treatment [20]. Several derivatives of 2’-FdC have shown promise as anti-HCV drugs with progress to clinical trials [31, 32]. However, due to their potential mitochondrial toxicity, the long-term adverse effects of treatment with 2’-deoxynucleoside analogues remains a concern [33]. Thus, although 2’-FdC is an interesting antiviral compound, its potential adverse effects as well as its combination with other compounds should be carefully evaluated in future studies.

In conclusion, 2’-FdC exerts potent anti-MNV effects in macrophages. Importantly, 2’-FdC acts synergistically with the well-known antivirals, including MPA, ribavirin, and T705. Although further studies are still required for evaluation of the antiviral effects of 2’-FdC or its derivatives against HuNV infection in robust models, our results suggest that 2’-FdC can serve as a potential backbone for anti-norovirus drug design.

## Electronic supplementary material

Below is the link to the electronic supplementary material.

Supplementary material 1 (DOCX 856 kb)
