# Supplementary material for: 2’-Fluoro-2’-deoxycytidine inhibits murine norovirus replication and synergizes MPA, ribavirin and T705
Source: Arch Virol. 2020 Aug 8;165(11):2605–13. doi: 10.1007/s00705-020-04759-4 (PMC7414258; doi:10.1007/s00705-020-04759-4)
Supplement: Supplementary file 1 — Supplementary material 1 (DOCX 856 kb) [file 705_2020_4759_MOESM1_ESM.docx]

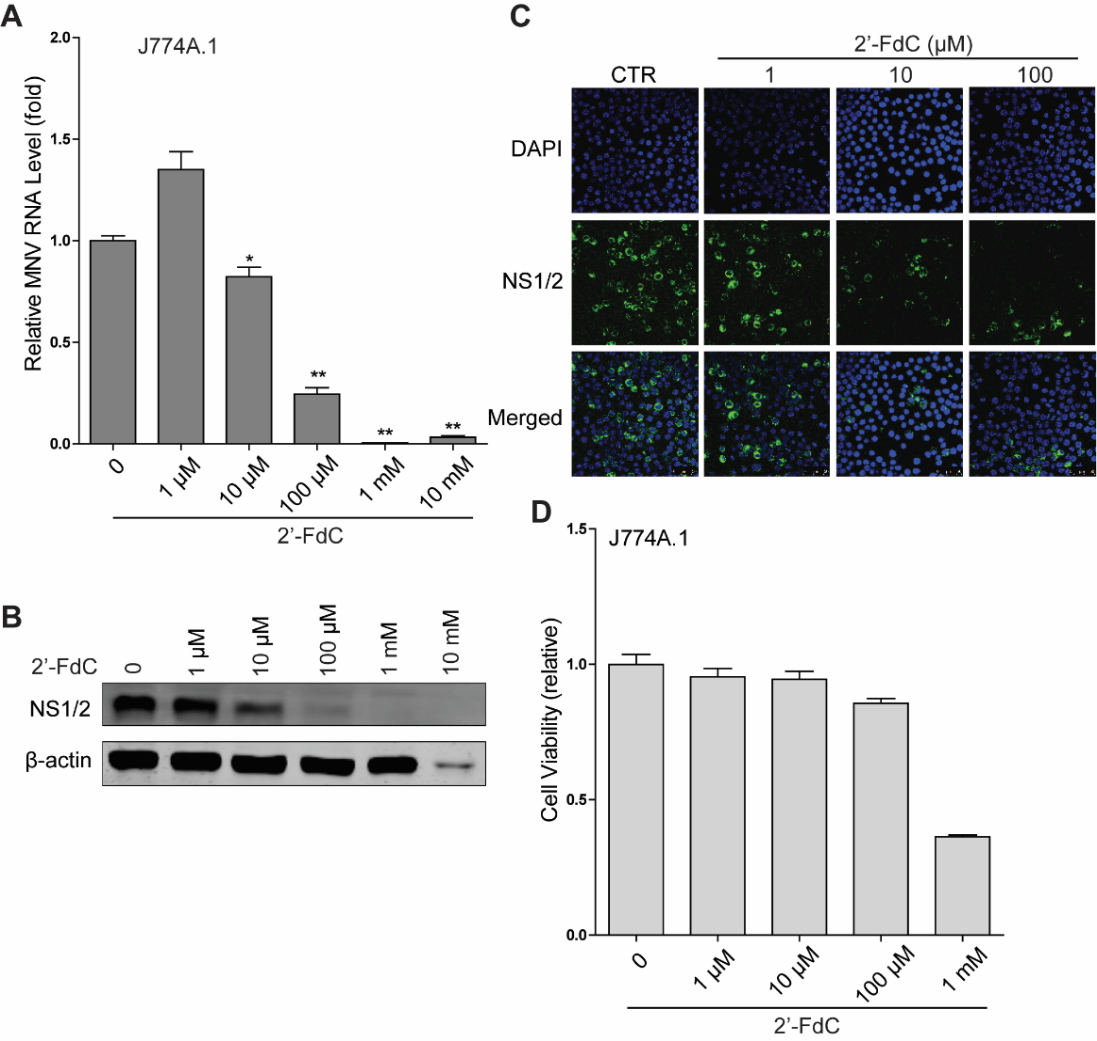


**Supplementary Fig. 1. 2’-FdC exerts anti-MNV activity in J774A.1 cells.** J774A.1 cells were infected with MNV-1 (MOI 1) for 1 h, then refreshed with culture medium containing different concentrations of 2’-FdC for 20 h. (A) The viral RNA level and (B) NS1/2 protein expression were analyzed by qRT-PCR (n = 5-6) and western blotting, respectively. (C) J774A.1 cells were infected with MNV-1 (MOI 1) for 1 h, then refreshed with culture medium containing different concentrations of 2’-FdC for 20 h. The viral NS1/2 protein expression was analyzed by confocal assay. (D) J774A.1 were untreated or treated with 2’-FdC with different concentrations for 20 h. The cytotoxicity was determined by MTT assay (n = 28-32). Data were normalized to the untreated control (set as 1). *P < 0.5; **P < 0.01. β-actin was used as a loading control.


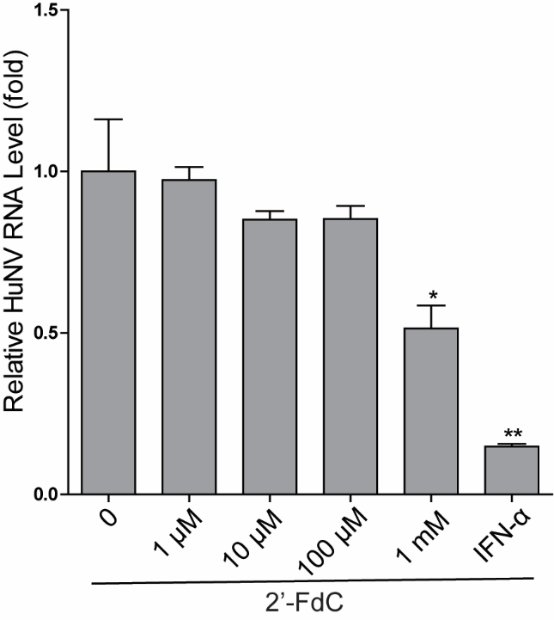


**Supplementary Fig. 2. The anti-HuNV ability of 2’-FdC in HG23 cells.** HG23 cells were untreated or treated with indicated concentrations of 2’-FdC or IFN-α (1000 IU/ml). After 2 days of treatment the antiviral activities were measured by qRT-PCR (n = 4-5). Data were normalized to the untreated control (set as 1). *P < 0.05; **P < 0.01.


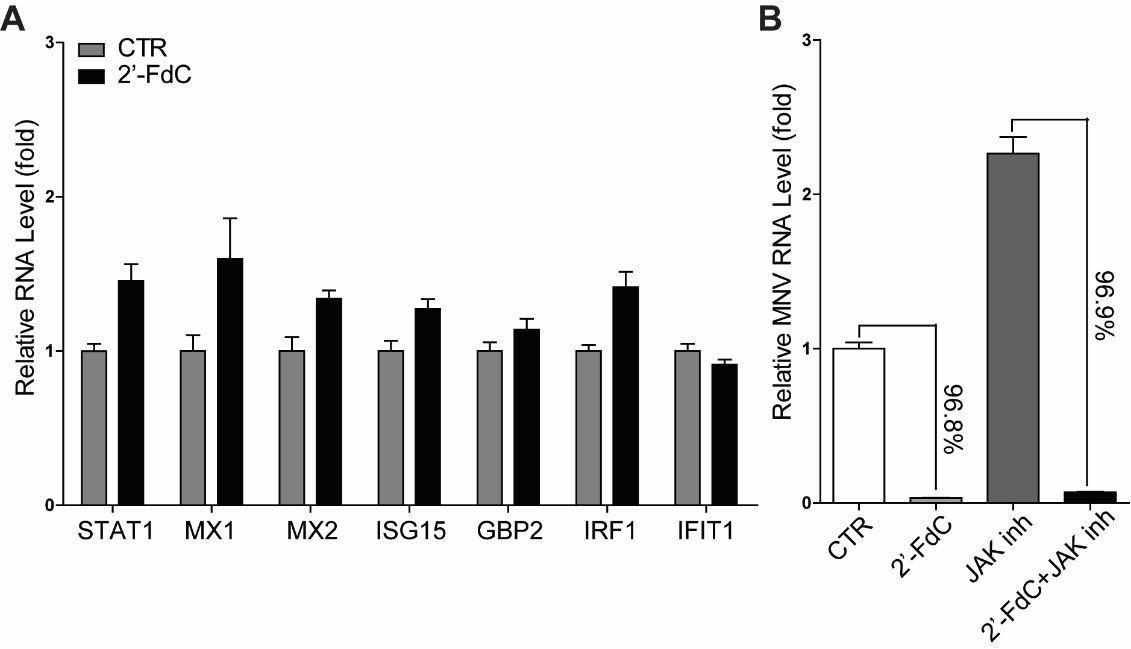


**Supplementary Fig. 3. The anti-MNV activity of 2’-FdC does not require ISG induction.** (A) RAW264.7 cells were untreated or treated with 2’-FdC (100 µM) for 20 h. The mRNA level of several ISGs were analyzed by qRT-PCR assay (n = 6). (B) RAW264.7 cells were infected with MNV-1 (MOI 1) for 1 h, then refreshed with culture medium containing 2’-FdC (100 µM), JAK inhibitor 1 (5 µg/ml) or combinations for 20 h. The viral RNA level was analyzed by qRT-PCR assay (n = 6). Data were normalized to the untreated control (set as 1).

**Supplementary Table 1. Primers used for qRT-PCR.**

| Gene | F-sequence (5’to 3’) | R-sequence (5’ to 3’) |
| --- | --- | --- |
| MNV-1 | CACGCCACCGATCTGTTCTG | GCGCTGCGCCATCACTC |
| mGAPDH | TTCCAGTATGACTCCACTCACGG | TGAAGACACCAGTAGACTCCACGAC |
| mSTAT1 | GCCTCTCATTGTCACCGAAGAAC | TGGCTGACGTTGGAGATCACCA |
| mISG15 | TGACGCAGACTGTAGACACG | TGGGGCTTTAGGCCATACTC |
| mMX1 | GGGGAGGAAATAGAGAAAATGAT | GTTTACAAAGGGCTTGCTTGCT |
| mMX2 | CCAGTTCCTCTCAGTCCCAAGATT | TACTGGATGATCAAGGGAACGTGG |
| mGBP2 | ACCAGCTGCACTATGTGACG | TCAGAAGTGACGGGTTTTCC |
| mIRF1 | CAGAGGAAAGAGAGAAAGTCC | CACACGGTGACAGTGCTGG |
| mIFIT1 | CCATAGCGGAGGTGAATATC | GGCAGGACAATGTGCAAGAA |
| hGAPDH | TGTCCCCACCCCCAATGTATC | CTCCGATGCCTGCTTCACTACCTT |
| HuNV^a^ | CGYTGGATGCGNTTYCATGA | CTTAGACGCATCATCATTYAC |

^a^ Y = C + T; R = A + G; W = A+T; N = any
